# Mesenchymal stem cell therapy in pulmonary fibrosis: a meta-analysis of preclinical studies

**DOI:** 10.1186/s13287-021-02496-2

**Published:** 2021-08-18

**Authors:** Deng-Yuan Li, Ru-Fang Li, Dan-Xiong Sun, Dan-Dan Pu, Yun-Hui Zhang

**Affiliations:** 1 Faculty of Life Science and Technology, Kunming University of Science and Technology, Kunming, 650500 People’s Republic of China; 2 The Affiliated Hospital of Kunming University of Science and Technology, Kunming, 650500 People’s Republic of China; 3 Department of Pulmonary and Critical Care Medicine, The First People’s Hospital of Yunnan Province, Kunming, 650022 People’s Republic of China

**Keywords:** Pulmonary fibrosis, Mesenchymal stem cell, Preclinical studies, Therapy, Meta-analysis

## Abstract

**Background:**

Pulmonary fibrosis (PF) is a devastating disease characterized by remodeling of lung architecture and abnormal deposition of fibroblasts in parenchymal tissue and ultimately results in respiratory failure and death. Preclinical studies suggest that mesenchymal stem cell (MSC) administration may be a safe and promising option in treating PF. The objective of our meta-analysis is to assess the efficacy of MSC therapy in preclinical models of PF.

**Methods:**

We performed a comprehensive literature search in PubMed, EMBASE, Web of Science, and Cochrane Library databases from inception to March 17, 2021. Studies that assessed the efficacy of MSC therapy to animals with PF were included. The SYRCLE bias risk tool was employed to evaluate the bias of included studies. The primary outcomes included survival rate and pulmonary fibrosis scores. Meta-analysis was conducted via Cochrane Collaboration Review Manager (version 5.4) and Stata 14.0 statistical software.

**Results:**

A total of 1120 articles were reviewed, of which 24 articles met inclusion criteria. Of these, 12 studies evaluated the survival rate and 20 studies evaluated pulmonary fibrosis scores. Compared to the control group, MSC therapy was associated with an improvement in survival rate (odds ratios (OR) 3.10, 95% confidence interval (CI) 2.06 to 4.67, *P* < 0.001, *I*^2^ = 0%) and a significant reduction in pulmonary fibrosis scores (weighted mean difference (WMD) 2.05, 95% CI −2.58 to −1.51, *P* < 0.001, *I*^2^ = 90%).

**Conclusions:**

MSC therapy is a safe and effective method that can significantly improve the survival and pulmonary fibrosis of PF animals. These results provide an important basis for future translational clinical studies.

**Supplementary Information:**

The online version contains supplementary material available at 10.1186/s13287-021-02496-2.

## Background

Pulmonary fibrosis (PF) is a chronic, life-threatening disease with a gradual worsening of pulmonary function and shortness of breath, and the median survival time of patients with idiopathic pulmonary fibrosis (IPF) was estimated to be 2.5–3.5 years [1, 2]. PF is characterized by alveolar epithelial cell injury, remodeling of lung architecture, abnormal accumulation of extracellular matrix, and fibroblasts in parenchymal tissue [3, 4], which ultimately results in respiratory failure and death [5]. The prognosis of IPF is poor, with a mortality rate comparable to advanced tumors [6]. In recent years, the treatments of medicine such as pirfenidone and nintedanib have improved lung function for patients with IPF [7], but neither one has a certain advantage on mortality outcomes, often necessitating lung transplantation [8, 9]. Therefore, it is important to detect innovative options and new therapeutic strategies for the management of pulmonary fibrosis.

In recent years, mesenchymal stem cells (MSCs) have received increasing attention in the field of regenerative applications, because of multi-lineage differentiation potential, migratory ability, and self-renewal properties [10, 11]. MSCs are derived from a variety of organs and tissues, such as the bone marrow and adipose tissues, and can home to the sites of injury. The therapeutic values of MSCs have been demonstrated in various diseases, including ischemic heart failure, pulmonary arterial hypertension, stroke, chronic kidney disease, and sepsis [12–15]. Accumulating evidence suggests the role of MSC administration in attenuating the disease by anti-apoptotic, immunomodulation, and anti-inflammatory effects [16–18]. However, the underlying molecular mechanisms are much more complicated and have not yet been fully recognized.

Several studies suggested that MSCs have the capacity to suppress inflammation, reduce fibrosis, and prolong the survival time for preclinical models of PF, which was induced by bleomycin, silica, paraquat (PQ), and radiation [10, 19–24]. However, the design projects, including MSC dose, type, route, source, and time interval, in each research are so different that the final therapeutic effect is difficult to evaluate. As a result, the best way of MSC therapy remains unclear. Therefore, we collected data from all relevant studies and conducted a meta-analysis to assess the efficacy of MSC treatment.

## Methods

### Data source and search strategies

This meta-analysis followed the Preferred Reporting Items for Systematic Reviews and Meta-Analyses (PRISMA) guidelines (Additional file 1: Table S1) [25]. A systematic literature search was performed using PubMed, EMBASE, Web of Science, and Cochrane Library from inception to March 17, 2021. We also manually reviewed the reference cited with the articles. The detailed search strategy is described in Additional file 2: Table S2. The language was limited to English.

### Eligible criteria

The following inclusion criteria were set: (1) the study involved animal models of pulmonary fibrosis (all species and sexes); (2) all pulmonary fibrosis animal models were subjected to MSC treatment; (3) studies that include efficacy outcomes, such as survival rate and pulmonary fibrosis scores; and (4) studies have a control group.

Studies were excluded from the meta-analysis for the following reasons: (1) all inclusion criteria were not fulfilled; (2) the MSCs used in the study were differentiated, or engineered to alter the expression of specific genes; (3) meeting abstracts, case reports, and case series; (4) review or meta-analysis; (5) the study was duplicated; and (6) studies published in a non-English language.

### Study selection and data extraction

Two investigators (Deng-Yuan Li and Ru-Fang Li) independently screened the literature according to the search strategy. Any disagreements were reviewed and resolved by a third investigator (Dan-Dan Pu and Dan-Xiong Sun). After identifying the articles that met the inclusion criteria and exclusion criteria, we extracted the data using a standardized collection form that included the first author, year of publication, animal characteristics (species, gender, and model), intervention details (origin, dose, route, and timing of MSC transplantation), and follow-up (observation time of outcomes after MSC therapy) and then measured the correlation with our primary outcomes (survival rate and pulmonary fibrosis scores). In the case of missing or unclear data for the primary outcome measures, an attempt was made to contact the author for clarification.

### Assessment of risk of bias

Risk of bias was assessed according to the Systematic Review Centre for Laboratory animal Experimentation (SYRCLE) bias risk tool [26]. The components included random sequence generation, performance bias, detection bias, attrition bias, reporting bias, and other sources of bias. For each item, studies were categorized as high, low, and unclear risk of bias.

### Primary outcomes

The main study outcomes of this meta-analysis were survival rate and pulmonary fibrosis scores [27].

### Statistical analysis

Statistical analysis was performed via Stata 14.0 statistical software and Cochrane Collaboration Review Manager (version 5.4). Continuous and dichotomous outcome variables were respectively described as weighted mean difference (WMD) and odds ratios (ORs) with 95% confidence intervals (CIs). The chi-squared test and *I*^2^ parameter were used to measure heterogeneity [28]. The fixed effects model was used for meta-analysis when *P* > 0.1 and *I*^2^ < 50%, and the random effects model was used when *P* < 0.1 and *I*^2^ > 50%. If heterogeneity was significant, subgroup analysis and meta-regression were performed to further exploration. We assessed the potential for publication bias using Funnel plots and Egger’s regression test [29]. Differences for which *P* < 0.05 (two-sided) were considered statistically significant.

## Results

### Study selection

**Fig. 1:**
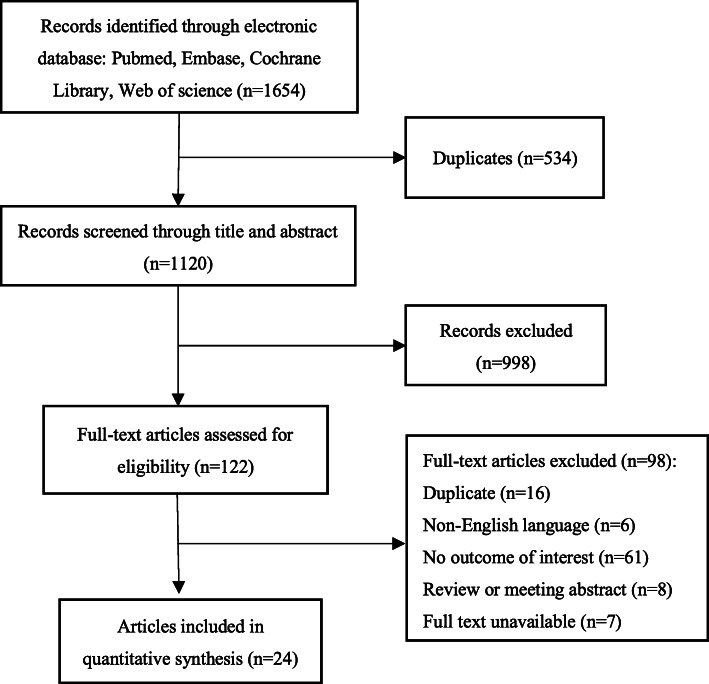
Flow diagram of the study selection

According to the search strategy, we identified 1654 studies related to mesenchymal stem cell therapies for pulmonary fibrosis, and 534 duplicate articles were removed using Endnote X 9 software. By reading the titles and abstracts, 122 articles were isolated for full-text review. Finally, 24 articles involving 564 animals were included in this meta-analysis after study selection (Fig. 1) (If more than one intervention was provided in a single study, each intervention was regarded as independent.).

### Study characteristics

The basic characteristics of 24 articles are listed in Table 1. The articles were published between 2005 and 2020. The majority of studies were conducted in rodents (rats and mice). One study was carried out in tree shrews. The pulmonary fibrosis animal model in most studies was induced by bleomycin, radiation, or paraquat. Stem cell types included bone marrow mesenchymal stem cells (BMSC) (*n* = 15), adipose-derived mesenchymal stem cells (ADMSC) (*n* = 6), umbilical cord mesenchymal stem cells (UCMSC) (*n* = 3), amniotic membrane mesenchymal stem cells (AMSC) (*n* = 2), human embryonic mesenchymal stem cells (EMSC) (*n* = 1), and human menstrual blood–derived mesenchymal stem cells (MenSC) (*n* = 1). The doses of interventions ranged from 10^3^ to 10^7^ MSCs, which were injected intravenously in 25 animal studies, via intraperitoneal transplantation in two studies, and intratracheal injection in one study. Timing of cell administration ranged from 1 h to 60 days after induction of the PF model. However, the majority of reports treated animals with stem cells prior to injury or evidence of PF. The duration of follow-up ranged from 1 to 60 days. Otherwise, three of the articles included multiple studies. Therefore, the meta-analysis included a total of 28 animal studies involving 564 animals.

**Table 1:** General characteristics of preclinical studies investigating the efficacy of MSC therapy in models of PF

Author (year)	Country	Species, strain, gender	No. of treated animals	No. of controls	PF model	MSC source	MSC dose	MSC route	Transplant type	Time of MSC therapy after PF	Follow-up (days)
Li et al. (2017) [43]	China	Mouse C57BL/6, M	5	5	Radiation	BMSC	2.0 × 10^6^	iv	Allograft	2 h	42 d
Xia et al. A (2015) [44]	China	Mouse, NR, NR	25	25	Radiation	BMSC	1.0 × 10^3^	iv	Xenograft	1 d	28 d
Xia et al. B (2015)	China	Mouse, NR, NR	25	25	Radiation	BMSC	5.0 × 10^3^	iv	Xenograft	1 d	28 d
Xia et al. C (2015)	China	Mouse, NR, NR	25	25	Radiation	BMSC	1.0 × 10^4^	iv	Xenograft	1 d	28 d
Guo et al. (2018) [23]	China	Tree shrews, NR, F	20	20	Radiation	UCMSC	3.0 × 10^7^	iv	Allograft	1 h, 7 d, 14 d, 21 d	28 d
F. Cahill et al. A (2016) [45]	Ireland	Mouse C57BL/6, F	5	5	BLM	BMSC	5.0 × 10^4^	iv	Allograft	6–8 h	28 d
F. Cahill et al. B (2016)	Ireland	Mouse C57BL/6, F	5	5	BLM	BMSC	5.0 × 10^4^	iv	Allograft	9 d	28 d
Zhang et al. (2018) [24]	China	Rat, SD, M	6	6	Silica	BMSC	2.0 × 10^6^	iv	Allograft	28 d	28 d
He et al. (2020) [19]	China	Rat, SD, M/F	22	20	PQ	AMSC	2.0 × 10^6^	iv	Xenograft	6 h	21 d
Moroncini et al. (2018) [46]	Italy	Mouse C57BL/6, F	8	8	BLM	UCMSC	2.5 × 10^5^	iv	Xenograft	1 d, 7 d	21 d
Chen et al. (2019) [10]	China	Mouse C57BL/6, M	10	10	PQ	BMSC	2.0 × 10^6^	iv	Allograft	7 d	14 d
Tashiro et al. (2015) [47]	USA	Mouse C57BL/6, M	5	12	BLM	ADMSC	5.0 × 10^5^	iv	Allograft	1 d	21 d
Ai et al. (2019) [48]	China	Mouse C57BL/6, M	10	10	BLM	ADMSC	5.0 × 10^6^	iv	Allograft	1 d	14 d
Zhang et al. (2019) [20]	China	Rat, SD, M	10	10	PQ	BMSC	3.0 × 10^6^	ip	Allograft	1 h	1 d
M. Kumamoto et al. (2009) [49]	Japan	Mouse C57BL/6, F	20	25	BLM	BMSC	5.0 × 10^6^	iv	Allograft	3 d	10 d
Reddy et al. (2016) [50]	India	Mouse Swiss-albino, M	10	10	BLM	ADMSC	4.0 × 10^7^	iv	Xenograft	3 d, 6 d, 9 d	21 d
Rojas et al. (2005) [51]	USA	Mouse C57BL/6, NR	5	6	BLM	BMSC	5.0 × 10^6^	iv	Allograft	6 h	14 d
Wang et al. (2012) [40]	China	Mouse BALB/c, M	6	6	BLM	EMSC	2.0 × 10^5^	iv	Xenograft	1 d	14 d
Lee et al. (2014) [31]	Korea	Mouse C57BL/6, M	10	20	BLM	ADMSC	3.0 × 10^5^	ip	Xenograft	60 d	60 d
Chen et al. (2018) [52]	China	Rat, SD, M	5	5	Silica	ADMSC	5.0 × 10^5^	iv	Allograft	1 d	28 d
Lee et al. (2010) [53]	Korea	Rat, SD, F	10	10	BLM	BMSC	1.0 × 10^7^	iv	Allograft	4 d	28 d
Periera-simon et al. (2020) [21]	USA	Mouse C57BL/6, NR	15	15	BLM	ADMSC	5.0 × 10^5^	iv	Xenograft	1 d	21 d
Aguilar et al. (2009) [22]	UK	Mouse C57BL/6, M	6	6	BLM	BMSC	5.0 × 10^6^ (1 d, 3 d)	iv	Allograft	8 h	14 d
Chen et al. (2020) [54]	China	Mouse C57BL/6, M	5	5	BLM	MenSC	5.0 × 10^5^	iv	Xenograft	2 d, 7 d	21 d
Lan et al. (2015) [38]	China	Mouse C57BL/6, F	6	6	BLM	BMSC	5.0 × 10^5^	it	Allograft	3 d	21 d
Yuben et al. (2009) [55]	Australia	Mouse, SCID, NR	8	8	BLM	UCMSC	1.0 × 10^6^	iv	Xenograft	1 d	28 d
Moodley et al. A (2013) [17]	Australia	Mouse C57BL/6, F	8	8	BLM	BMSC	1.0 × 10^6^	iv	Xenograft	3 d	21 d
Moodley et al. B (2013)	Australia	Mouse C57BL/6, F	8	8	BLM	AMSC	1.0 × 10^6^	iv	Xenograft	3 d	21 d

*PF* pulmonary fibrosis, *SD* Sprague Dawley, *M* male, *F* female, *NR* not reported, *MSC* mesenchymal stem cell, *BLM* bleomycin, *PQ* paraquat, *BMSC* bone marrow mesenchymal stem cells, *UCMSC* umbilical cord mesenchymal stem cells, *ADMSC* adipose-derived mesenchymal stem cells, *EMSC* human embryonic mesenchymal stem cells, *AMSC* amniotic mesenchymal stem cells, *MenSC* human menstrual blood–derived mesenchymal stem cells, *iv* intravenous, *ip* intraperitoneally, *it* intratracheally. Follow-up (days) suggests the observation time of outcomes after mesenchymal stem cell administration

### Risk of bias (SYRCLE tool)

**Fig. 2:**
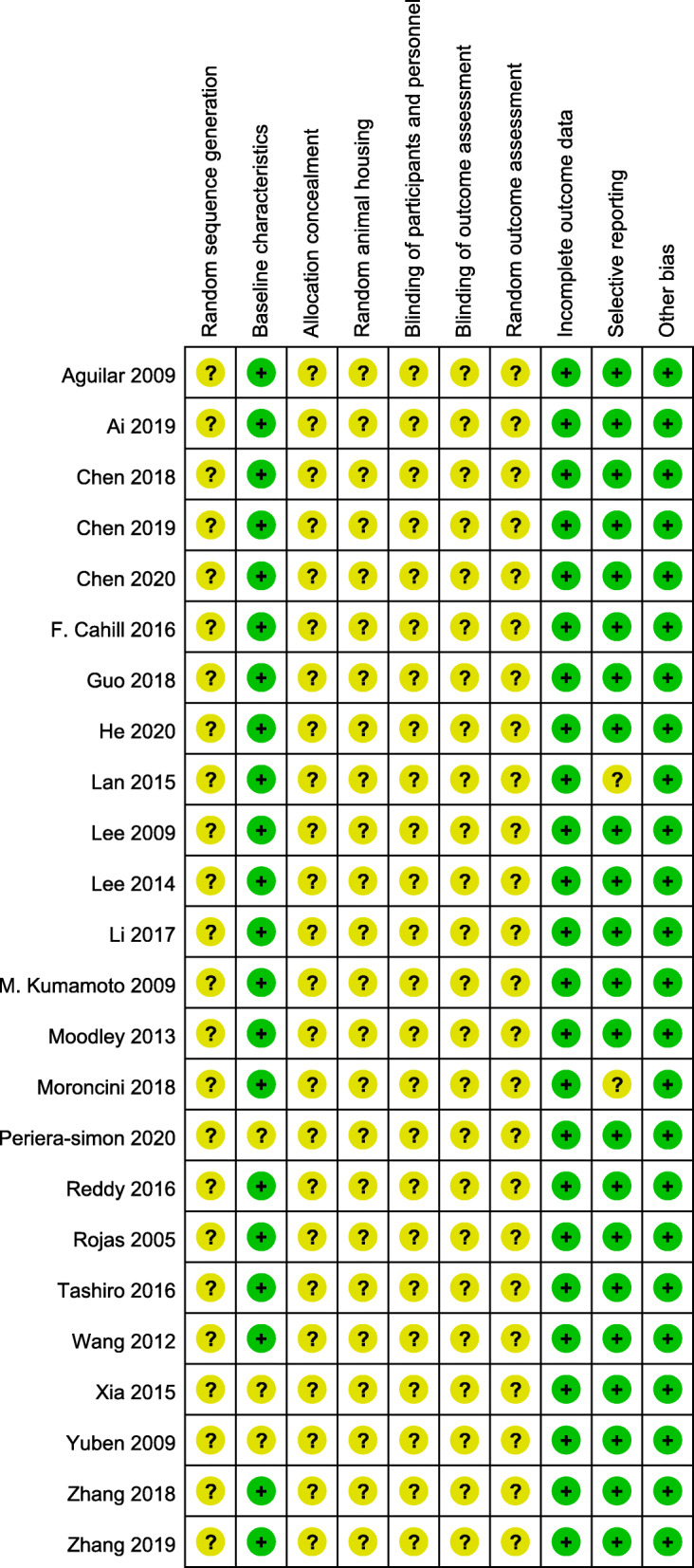
Risk of bias assessment using the SYRCLE tool

Each risk of bias item of all articles is shown in Fig. 2. No study fulfilled all ten criteria for low risk of bias. Most studies demonstrated similar baseline characteristics about experimental and control groups. Among the 24 articles, none of the studies accurately described the random sequence generation. Therefore, the risk of bias in the randomization sequence was judged to be “unclear.” In addition, because of the special properties of mesenchymal stem cell administration, it is difficult for researchers to achieve a blinding procedure when acquiring stem cells, although this does not influence the experimental results. The majority of studies were scored as having a low risk of reporting and attrition bias. The risk of bias was unclear for all articles across the domains of allocation concealment, random animal housing, and random outcome assessment. Moreover, no additional sources of bias were identified.

### Efficacy of MSC therapy on PF

#### Survival rate

**Fig. 3:**
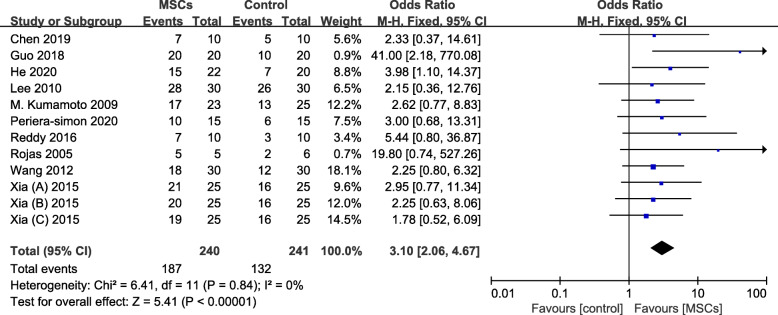
Forest plot showing the effect of MSC therapy on survival rate

In this meta-analysis, a total of 12 animal studies reported survival rate. A fixed effects model was used to assess this research, as the heterogeneity was low (*I*^2^ = 0% and *P* = 0.84). Animals treated with MSCs had a significantly increasing survival rate compared to control (OR 3.10, 95% CI 2.06 to 4.67, *P* < 0.001) (Fig. 3).

#### Pulmonary fibrosis scores

**Fig. 4:**
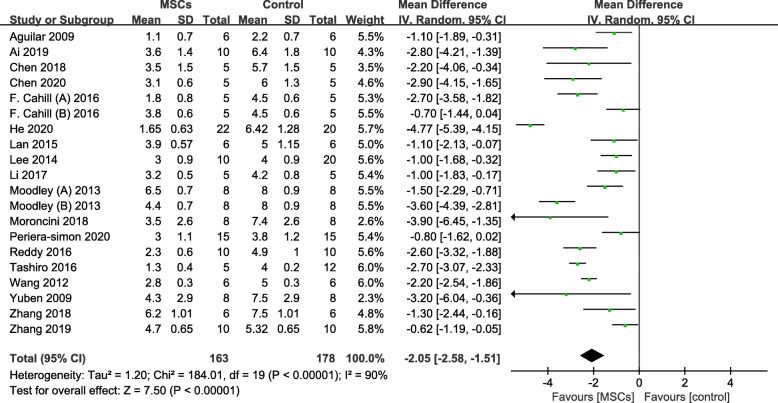
Forest plot showing the effect of MSC therapy on pulmonary fibrosis scores

The results of pulmonary fibrosis scores in this meta-analysis are shown in Fig. 4. The pooled weighted mean difference (WMD) for pulmonary fibrosis scores was −2.05 (95% CI −2.58 to −1.51), and the *P* values were less than 0.001, which demonstrated that MSC therapy was associated with an obvious reduction in pulmonary fibrosis scores compared with that in the control group. The significant heterogeneity was detected in this study (*I*^2^ = 90% and *P* < 0.01); thus, a random effects model was used.

### Subgroup analysis and meta-regression analysis

We analyzed the source of heterogeneity by evaluating pulmonary fibrosis scores in a subgroup analysis (Table 2, Additional file 3: Fig. S1-S7). We focused on MSC type, dose, time, injection route, type of graft, PF model, and geographic location, which were reported in tables, respectively. The composite WMDs (95% CI) for the PF model (silica-induced) and MSC type (BMSC) were −1.55 (−2.52 to −0.57), *I*^2^ = 0%, and −1.22 (−1.68 to −0.76), *I*^2^ = 60% (*P* < 0.01), respectively. With regard to MSC dose, the composite WMDs (95% CI) for high-dose BMSC and high-dose ADMSC were −1.00 (−1.42 to −0.58), *I*^2^ = 14%, and −2.46 (−3.28 to −2.00), *I*^2^ = 0%, respectively (*P* < 0.01) (Additional file 3: Fig. S8-S9).

**Table 2:** The study of correlation grouping and heterogeneity between pulmonary fibrosis scores and variables

Subgroup	Weighted mean (95% CI)	***I***^***2***^	***P***
**PF model**			
Bleomycin-induced PF model	−2.03 (−2.51, −1.56)	82%	< 0.01
Silica-induced PF model	−1.55 (−2.52, −0.57)	0%	< 0.01
Paraquat-induced PF model	−2.69 (−6.76, 1.37)	99%	< 0.01
Radiation-induced PF model	−1.00 (−1.83, −0.17)	NA	0.02
**MSC route**			
Intravenous injection	−2.77 (−2.83, −1.71)	88%	< 0.01
Non-intravenous injection	−0.83 (−1.23, −0.42)	0%	0.6
**MSC type**			
BMSC	−1.22 (−1.68, −0.76)	60%	< 0.01
ADMSC	−1.98 (−2.79, −1.17)	84%	< 0.01
Others	−3.41 (−4.61, −2.22)	91%	< 0.01
**MSC dose**			
< 1.0 × 10^6^ MSCs	−1.79 (−2.33, −1.25)	83%	< 0.01
≥ 1.0 × 10^6^ MSCs	−2.34 (−3.45, −1.23)	93%	< 0.01
**Time of MSC therapy**			
≤ 1 d	−2.21 (−2.96, −1.47)	92%	< 0.01
>1 d	−1.83 (−2.59, −1.07)	84%	< 0.01
**Type of graft**			
Allograft	−1.58 (−2.25, −0.91)	86%	< 0.01
Xenograft	−2.55 (−3.40, −1.70)	91%	< 0.01
**Geographic location**			
Asia	−2.03 (−2.84, −1.23)	92%	< 0.01
Europe	−1.79 (−2.95, −0.64)	81%	< 0.01
America	−1.79 (−3.65, −0.07)	94%	< 0.01
Oceania	−2.68 (−4.38, −0.98)	85%	< 0.01

To further identify the potential heterogeneity across studies, we estimated the effect of all variables on the study results by using meta-regression. For pulmonary fibrosis scores, MSC type remained the only significant factor (*P* = 0.008), indicating that MSC type may be the source of heterogeneity in this meta-analysis (Additional file 4: Table S3).

### Sensitivity analyses

Sensitivity analyses were performed using “one-study-removed” analyses. We used sensitivity analyses to evaluate whether the pooled effect size still fell within the total pooled effect size of the 95% CI, indicating that the outcomes of the meta-analysis were stable (Additional file 5: Fig. S10).

### Publication bias

Funnel plots and Egger’s regression tests were used to assess publication bias in survival rate and pulmonary fibrosis scores individually (Additional file 6: Fig. S11). The Funnel plots and Egger’s regression tests (*P* = 0.015) showed publication bias in survival rate and no significant publication bias in pulmonary fibrosis scores (*P* = 0.702).

## Discussion

This meta-analysis evaluated the efficacy of MSC therapy in preclinical models of pulmonary fibrosis. In general, the results of our meta-analysis indicate an improvement in lung damage with MSC treatment, which is consistent with the previous meta-analysis [30]. While this previous meta-analysis only included the animal model of PF induced by bleomycin, we updated another three PF models, including silica, paraquat, and radiation, and analyzed another important parameter (survival rate), for providing more possibilities for MSC therapy in preclinical studies of PF.

In our research, the majority of MSCs were derived from fresh tissue of healthy rodents or humans. The culture medium was changed every 2–3 days until cell confluency reached > 80%. Generations 2–5 of the MSCs were harvested for the identification and cell administration studies [10, 23, 31]. In the field of regenerative medicine, animal studies are relevant to clinical application and used to evaluate the safety and efficacy of MSC therapy. As far as we know, several clinical trials have been conducted to explore the potential benefits of MSC transplantation for patients with IPF [32–36]. A phase 1b clinical trial reported that the intravenous route of up to 2 × 10^6^ placental MSCs per kilogram is safe in patients with moderately severe IPF. They found that this intervention was not associated with significant adverse reactions and that lung function, 6-min walk distance (6MWD), and computed tomography (CT) fibrosis score were unchanged over 6 months compared with baseline [35]. Another clinical trial indicated a total dose of 1.6 × 10^9^ MSCs is well tolerated by IPF patients with a rapid lung function decline, which can improve 6MWD in 13 weeks and lung function in 39 weeks [36]. Even though MSC therapy is a generally safe and promising candidate to slow the disease progression in these clinical trials, the best way of MSC therapy remains unclear.

In our meta-analysis, MSC treatment obviously improved the survival rate and fibrosis scores of PF animal models, indicating the potentials of MSC transplantation in preclinical studies of PF. We investigated the efficacy of different MSC sources and found that the most common type of employed stem cells was BMSC or ADMSC (54/21%). However, our study reported that other types of MSC (AMSC, UCMSC, EMSC, and MenSC) showed better treatment results in regard to pulmonary fibrosis scores. Due to the lack of related reports, more rigorous studies treated with MSCs are required to verify these findings. In addition, recent studies involving overexpressing specific genes [37] in engrafted stem cells or preconditioned MSCs have shown improvement on survival rate and lung fibrosis following MSC administration [38–40]. In fact, confirmatory studies of two models could improve the validity; however, we did not collect and identify any studies regarding stem cell therapy in gene modification.

We attempt to explore the heterogeneity from different design projects, including different stem cell types, injection routes, time intervals, and dosages of MSCs. According to subgroup analysis and meta-regression, we found that the possible contributor of heterogeneity is the PF model, MSC type, and MSC dose. There are many interventions that can induce pulmonary fibrosis, including bleomycin, silica, paraquat, radiation, and so on, which have their advantages and disadvantages. The pathological features of the PF model diverge in many aspects may be the reason for the different therapeutic effects. Migrated MSCs play an important function in injury repair by differentiating into different tissue, anti-apoptotic, immunomodulation, and anti-inflammatory effects [18]. At the same time, the adverse environment around the damaged lung may also affect the efficacy of implanted MSCs [41].

The success of MSC therapy is partly reliant on a sufficient number of cells reaching the target organ and appropriate timing of stem cell injection. In murine studies, the effective dose for stem cell treatment is normally 1.0 × 10^6^ per 30 g mouse. And the early transplantation of MSCs within 1 day after lung damage is the most valuable method for repairing fibrotic sites [42]. Subgroup analyses were performed according to the dosage of MSCs (< 1.0 × 10^6^ MSCs or ≥ 1.0 × 10^6^ MSCs) and time of MSC therapy (≤ 1 day or >1 day) and indicated that early stem cell therapy and high dose of MSCs in the experimental group appear to be more effective than in the control group. However, the majority of reports treated animals with stem cells prior to injury or evidence of PF, suggesting that MSC therapy does not show a significant effect on the subsequent collagen deposition and fibrosis prevention when administrating few days after the lung damage. The intravenous injection had a greater effect size than other routes, demonstrating that this method had better therapeutic value, although the effect sizes remained large for other routes also. Furthermore, xenograft therapy in PF animals was more effective than allograft therapy, implying that graft type is a crucial factor in clinical application for PF therapy. However, the sample size of this subgroup analysis was small, and there may be false-positive or false-negative conclusions.

The SYRCLE Risk of Bias tool was used to assess the translational potentials of MSC therapy [26]. In this meta-analysis, none of 24 articles was identified as having a low risk of bias according to the reporting contents in this tool. Even though most of the studies tried to avoid all kinds of bias, few researchers attempt to show their protocols. Consequently, we could not estimate the effect size for follow-up studies based on this tool. The low quality of methodology was mainly caused by inappropriate sequence generation, lack of double-blinding and allocation concealment, selective reporting, and incomplete outcome data. This meta-analysis highlights the common problems and recommends an urgent need for higher methodological quality when publishing. To reduce the risk of bias and take the poor reporting of outcome measures into consideration, we suggest that future translational studies related to MSC-based PF treatment should follow the SYRCLE Risk of Bias tool and report both detailed methodology and measures’ performance.

There are several strengths in our meta-analysis. Firstly, this study for the first time evaluated the survival rate on the PF model in preclinical research. Even though a previous meta-analysis evaluated the benefits of MSCs in a BLM-induced animal model, the meta-analysis reported here incudes different PF models and recently published high-quality studies. Secondly, we performed a systematic literature search, comprehensive data collection, and subgroup analysis by MSC type, injection route, and timing, which can improve the accuracy of our findings. Thirdly, the main results about survival rate and pulmonary fibrosis scores could provide vital insight into the future study.

However, our study also has some limitations. Firstly, the funnel plots and Egger’s regression tests detected that publication bias exists in this meta-analysis. As expected, studies reporting positive results are easier to publish, especially in animal studies. Secondly, the included studies were limited to those that had been published. The outcomes will be altered when undocumented data are published. Thirdly, current data mostly focus on the effect of MSC administration prior to injury or evidence of PF, while results of late administration are limited. Whether MSC therapy can significantly alleviate pulmonary fibrosis and produce long-term therapeutic effects on regeneration is worth further exploration. Fourthly, the diagnostic approach to PF is reliant on high-resolution chest computed tomography (HRCT), pulmonary function tests, and histologic findings [1]. Among them, HRCT and pulmonary function tests are more conductive to clinical application. However, most of the studies in our meta-analysis did not use HRCT and lung function for diagnosis. Therefore, it is suggested that future preclinical studies should concentrate on MSC transplantation at a more advanced stage and non-invasive diagnosis.

## Conclusion

In conclusion, this meta-analysis evaluated the efficacy of mesenchymal stem cell therapy on survival rate and pulmonary fibrosis scores in animal models, which provides an important basis for future translational clinical studies. Due to the low methodological quality, large sample, prospective, double-blind, randomized controlled trials are required to prove the safety and efficacy of MSC therapy for IPF.

## Supplementary Information

**Additional file 1: Table S1.** PRISMA 2009 Checklist.

**Additional file 2: Table S2.** The detailed search strategy.

**Additional file 3: Figure S1.** Forest plot summarizing the relationship between PF models and pulmonary fibrosis scores in preclinical models of PF. Figure S2. Forest plot summarizing the relationship between MSC type and pulmonary fibrosis scores in preclinical models of PF. **Figure S3.** Forest plot summarizing the relationship between MSC dose and pulmonary fibrosis scores in preclinical models of PF. Figure S4. Forest plot summarizing the relationship between MSC route and pulmonary fibrosis scores in preclinical models of PF. **Figure S5.** Forest plot summarizing the relationship between transplant type and pulmonary fibrosis scores in preclinical models of PF. **Figure S6.** Forest plot summarizing the relationship between timing of MSC therapy after PF and pulmonary fibrosis scores in preclinical models of PF. **Figure S7.** Forest plot summarizing the relationship between geographic location and pulmonary fibrosis scores in preclinical models of PF. **Figure S8.** Forest plot summarizing the relationship between BMSC dose and pulmonary fibrosis scores in preclinical models of PF. **Figure S9.** Forest plot summarizing the relationship between ADMSC dose and pulmonary fibrosis scores in preclinical models of PF.

**Additional file 4: Table S3.** Meta-regression showing the possible source of heterogeneity of meta-analysis.

**Additional file 5: Fig. S10.** Sensitivity analyses: a) survival rate, b) pulmonary fibrosis scores.

**Additional file 6: Fig. S11.** Funnel plots for (a) survival rate, (b) pulmonary fibrosis scores.

## Data Availability

All supporting data are included in the article and its additional files.

## Abbreviations

MSC: Mesenchymal stem cell
IPF: Idiopathic pulmonary fibrosis
SYRCLE: Systematic Review Centre for Laboratory Animal Experimentation
PQ: Paraquat
WMD: Weighted mean difference
