# Supplementary material for: Mesenchymal stem cell therapy in pulmonary fibrosis: a meta-analysis of preclinical studies
Source: Stem Cell Res Ther. 2021 Aug 18;12:461. doi: 10.1186/s13287-021-02496-2 (PMC8371890; doi:10.1186/s13287-021-02496-2)
Supplement: Supplementary file 2 — Additional file 2: Table S2. The detailed search strategy. [file 13287_2021_2496_MOESM2_ESM.docx]

**Table S2. The detailed search strategy**

| **Electronic databases** | **Search** | **Search strategy** |  | **Results** |
| --- | --- | --- | --- | --- |
| **PUBMED** | #1 | "pulmonary fibrosis"[Title/Abstract] OR "idiopathic pulmonary fibrosis"[Title/Abstract] OR "Asbestosis"[Title/Abstract] OR "Pneumoconiosis"[Title/Abstract] |  | 27478 |
|  | #2 | "Pulmonary Fibrosis"[MeSH Terms] OR "Idiopathic Pulmonary Fibrosis"[MeSH Terms] OR "Asbestosis"[MeSH Terms] OR "Pneumoconiosis"[MeSH Terms] |  | 43574 |
|  | #3 | #1 OR #2 |  | 52370 |
|  | #4 | "mesenchymal stem cell*"[Title/Abstract] OR "mesenchymal stromal cells"[Title/Abstract] OR "mesenchymal stromal cell"[Title/Abstract] OR "bone marrow mesenchymal stem cell*"[Title/Abstract] OR "bone marrow stromal cell*"[Title/Abstract] OR "bone marrow stromal stem cells"[Title/Abstract] OR "multipotent bone marrow stromal cell*"[Title/Abstract] OR "adipose derived mesenchymal stem cell*"[Title/Abstract] OR "adipose tissue derived mesenchymal stem cell*"[Title/Abstract] OR "adipose tissue derived mesenchymal stromal cells"[Title/Abstract] OR "adipose derived mesenchymal stromal cells"[Title/Abstract] OR "adipose derived mesenchymal stem cell"[Title/Abstract] OR "multipotent mesenchymal stromal cell*"[Title/Abstract] OR "mesenchymal progenitor cell*"[Title/Abstract] OR "wharton jelly cells"[Title/Abstract] OR "wharton s jelly cell*"[Title/Abstract] OR "whartons jelly cells"[Title/Abstract] |  | 62268 |
|  | #5 | "mesenchymal stem cells"[MeSH Terms] OR "mesenchymal stem cell transplantation"[MeSH Terms] |  | 43648 |
|  | #6 | #4 OR #5 |  | 69384 |
|  | #7 | #3 AND #6 |  | **346** |
| **EMBASE** | #1 | ' pulmonary fibrosis ':ab,ti |  | 320594 |
|  | #2 | ' pulmonary fibrosis '/exp |  | 90565 |
|  | #3 | #1 OR #2 |  | 94157 |
|  | #4 | ' mesenchymal stem cells ':ab,ti |  | 60364 |
|  | #5 | ' mesenchymal stem cells '/exp |  | 64496 |
|  | #6 | #4 OR #5 |  | 77600 |
|  | #7 | #3 AND #6 |  | **628** |
| **Cochrane Library** | #1 | (pulmonary fibrosis):ti,ab,kw |  | 3435 |
|  | #2 | MeSH descriptor: [Pulmonary Fibrosis] explode all trees |  | 564 |
|  | #3 | #1 or #2 |  | 3442 |
|  | #4 | (mesenchymal stem cells):ti,ab,kw |  | 1574 |
|  | #5 | MeSH descriptor: [Mesenchymal Stem Cells] explode all trees |  | 112 |
|  | #6 | #4 or #5 |  | 1574 |
|  | #7 | #3 and #6 |  | **17** |
| **Web of Science** | #1 | TOPIC: (" pulmonary fibrosis ") |  | 31171 |
|  | #2 | TOPIC:(" mesenchymal stem cells ") |  | 84723 |
|  | #3 | #1 AND #2 |  | **663** |
