# Supplementary material for: Mesenchymal stem cell therapy in pulmonary fibrosis: a meta-analysis of preclinical studies
Source: Stem Cell Res Ther. 2021 Aug 18;12:461. doi: 10.1186/s13287-021-02496-2 (PMC8371890; doi:10.1186/s13287-021-02496-2)
Supplement: Supplementary file 3 — Additional file 3: Figure S1. Forest plot summarizing the relationship between PF models and pulmonary fibrosis scores in preclinical models of PF. Figure S2. Forest plot summarizing the relationship between MSC type and pulmonary fibrosis scores in preclinical models of PF. Figure S3. Forest plot summarizing the relationship between MSC dose and pulmonary fibrosis scores in preclinical models of PF. Figure S4. Forest plot summarizing the relationship between MSC route and pulmonary fibrosis scores in preclinical models of PF. Figure S5. Forest plot summarizing the relationship between transplant type and pulmonary fibrosis scores in preclinical models of PF. Figure S6. Forest plot summarizing the relationship between timing of MSC therapy after PF and pulmonary fibrosis scores in preclinical models of PF. Figure S7. Forest plot summarizing the relationship between geographic location and pulmonary fibrosis scores in preclinical models of PF. Figure S8. Forest plot summarizing the relationship between BMSC dose and pulmonary fibrosis scores in preclinical models of PF. Figure S9. Forest plot summarizing the relationship between ADMSC dose and pulmonary fibrosis scores in preclinical models of PF. [file 13287_2021_2496_MOESM3_ESM.docx]

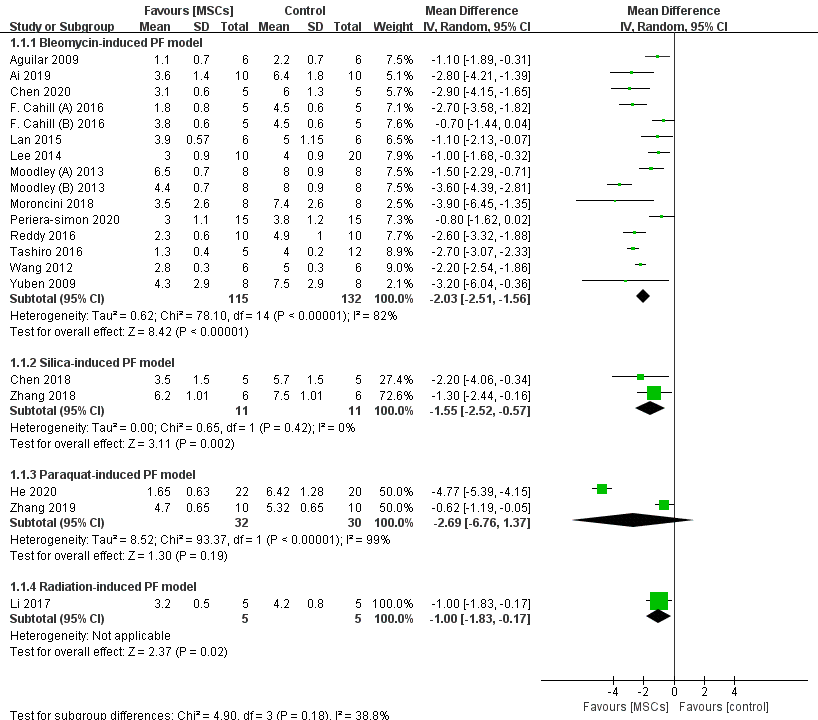


Fig. S1 Forest plot summarizing the relationship between PF models and pulmonary fibrosis scores in preclinical models of PF.


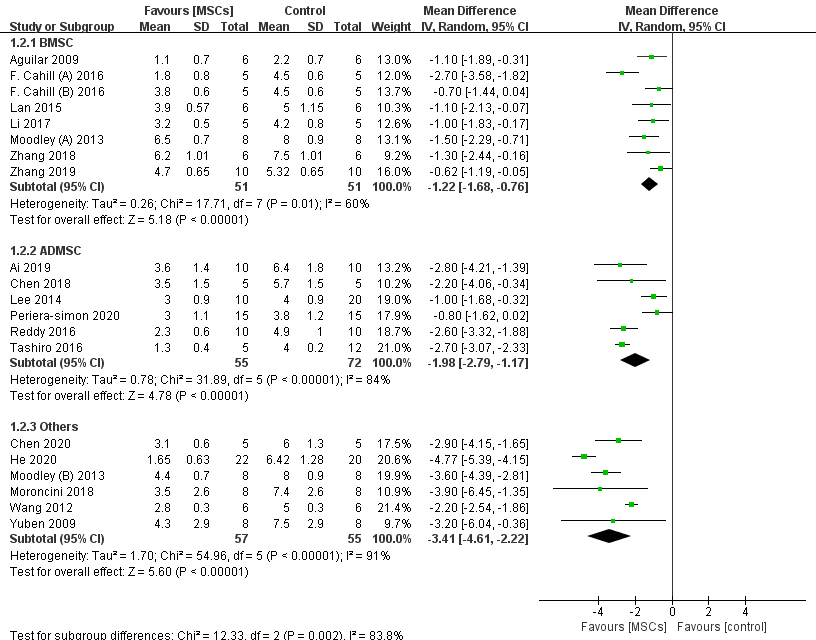


Fig. S2 Forest plot summarizing the relationship between MSCs type and pulmonary fibrosis scores in preclinical models of PF.


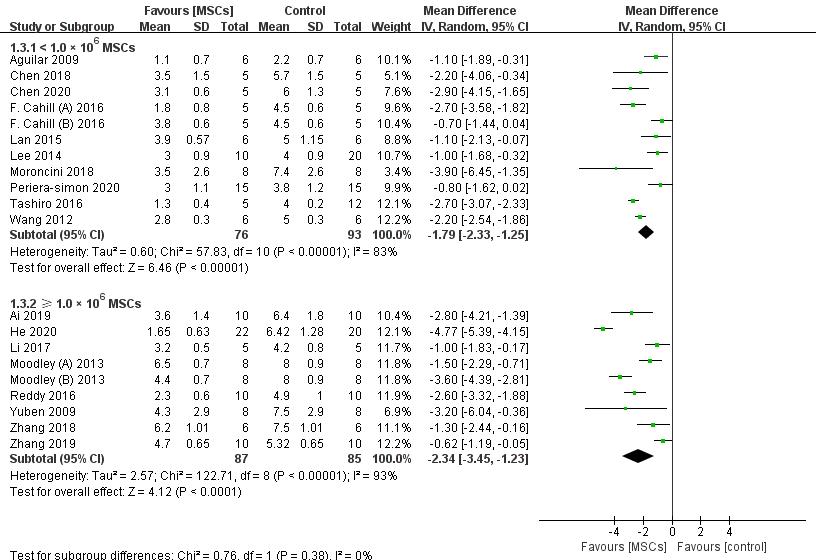


Fig. S3 Forest plot summarizing the relationship between MSCs dose and pulmonary fibrosis scores in preclinical models of PF.


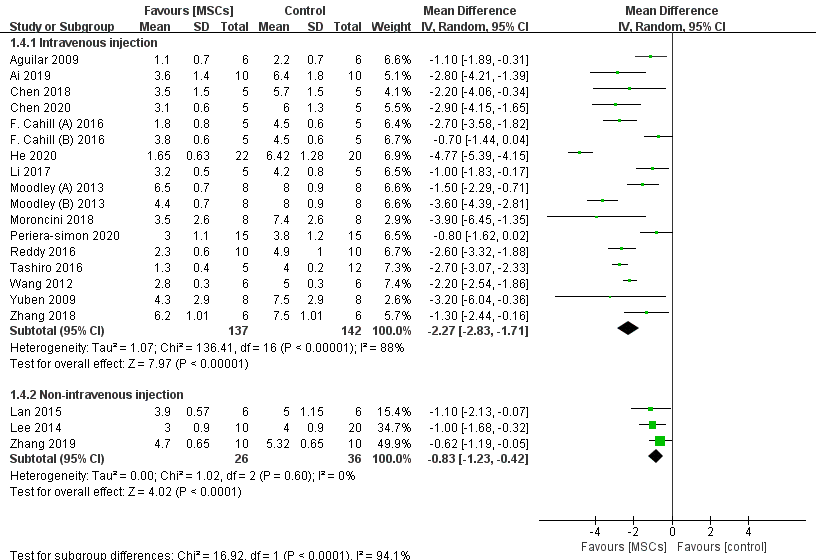


Fig. S4 Forest plot summarizing the relationship between MSCs route and pulmonary fibrosis scores in preclinical models of PF.


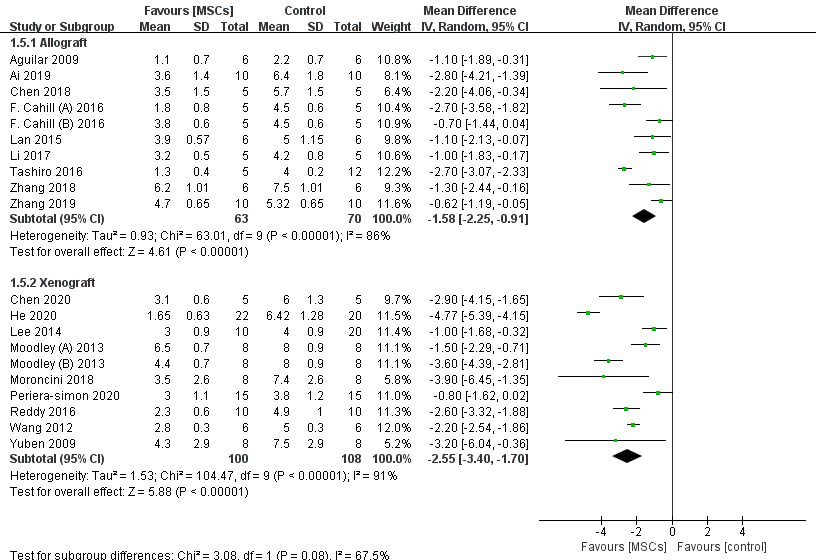


Fig. S5 Forest plot summarizing the relationship between transplant type and pulmonary fibrosis scores in preclinical models of PF.


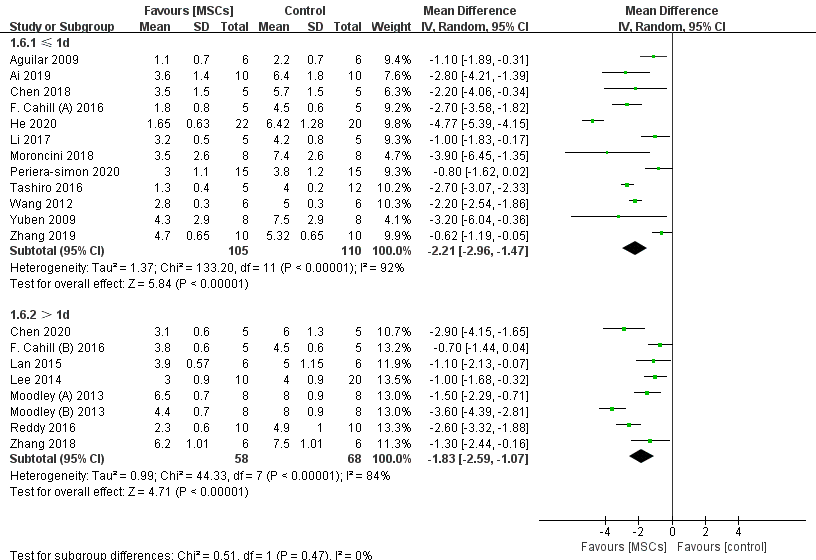


Fig. S6 Forest plot summarizing the relationship between timing of MSCstherapy after PF and pulmonary fibrosis scores in preclinical models of PF.


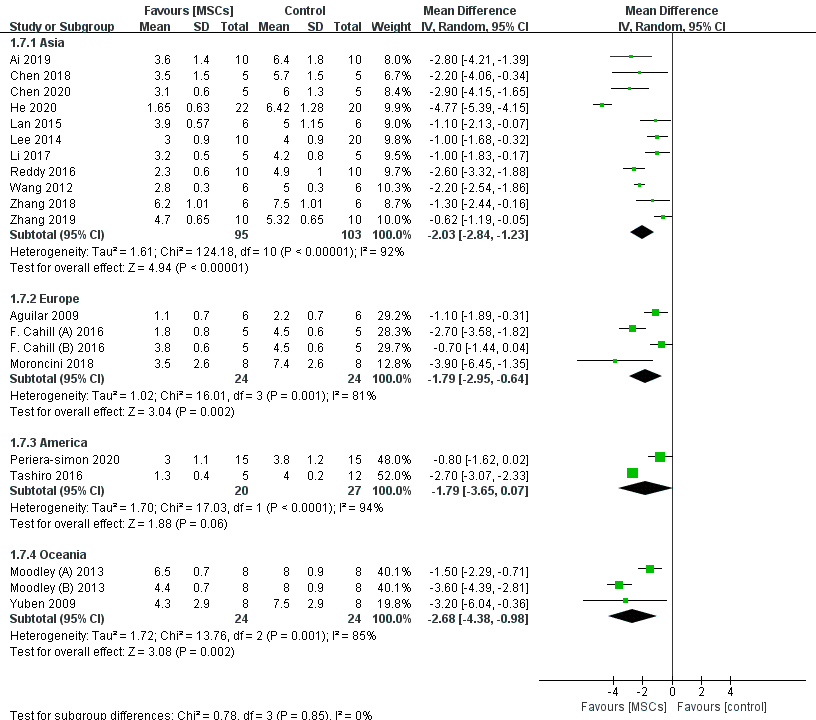


Fig. S7 Forest plot summarizing the relationship between geographic location and pulmonary fibrosis scores in preclinical models of PF.


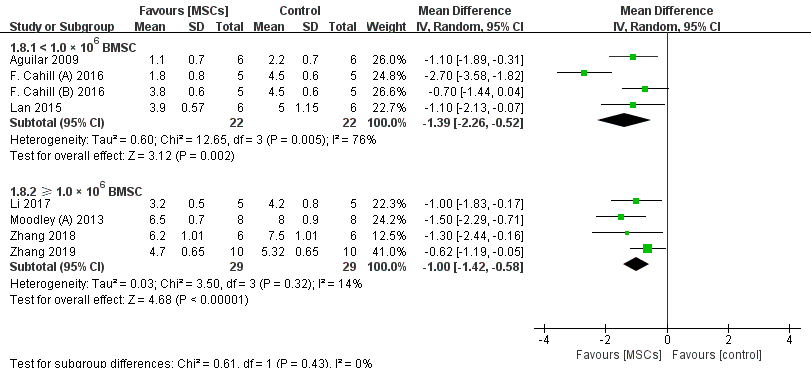


Fig. S8 Forest plot summarizing the relationship between BMSC dose and pulmonary fibrosis scores in preclinical models of PF.


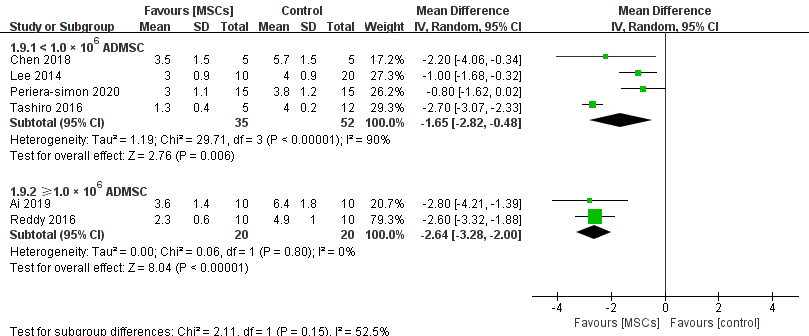


Fig. S9 Forest plot summarizing the relationship between ADMSC dose and pulmonary fibrosis scores in preclinical models of PF.
