# Supplementary material for: Mesenchymal stem cell therapy in pulmonary fibrosis: a meta-analysis of preclinical studies
Source: Stem Cell Res Ther. 2021 Aug 18;12:461. doi: 10.1186/s13287-021-02496-2 (PMC8371890; doi:10.1186/s13287-021-02496-2)
Supplement: Supplementary file 4 — Additional file 4: Table S3. Meta-regression showing the possible source of heterogeneity of meta-analysis. [file 13287_2021_2496_MOESM4_ESM.docx]

Table S3. Meta-regression showing the possible source of heterogeneity of meta-analysis.

| **Variables** | **Coef** | **SE** | ***P*** | **95%CI** |
| --- | --- | --- | --- | --- |
| PF models | 0.15 | 0.37 | 0.695 | (-0.66, 0.96) |
| MSCs route | 0.61 | 0.62 | 0.342 | (-0.73, 1.96) |
| MSCs type | -1.29 | 0.41 | 0.008 | (-2.18 -0.40) |
| MSCs dose | -0.78 | 0.58 | 0.203 | (-2.04, 0.48) |
| Time of MSCs therapy after PF | 0.48 | 0.54 | 0.931 | (-1.14, 1.23) |
| Transplant type | -0.77 | 0.68 | 0.279 | (-0.71, 2.24) |
| Geographic location | -0.11 | 0.23 | 0.964 | (-0.52, 0.50) |

*Coef* coefficient*, SE* standard error.
