# Supplementary material for: Mesenchymal stem cell therapy in pulmonary fibrosis: a meta-analysis of preclinical studies
Source: Stem Cell Res Ther. 2021 Aug 18;12:461. doi: 10.1186/s13287-021-02496-2 (PMC8371890; doi:10.1186/s13287-021-02496-2)
Supplement: Supplementary file 5 — Additional file 5: Fig. S10. Sensitivity analyses: a) survival rate, b) pulmonary fibrosis scores. [file 13287_2021_2496_MOESM5_ESM.docx]

a

b

Fig. S10 Sensitivity analyses: a) survival rate, b) pulmonary fibrosis scores.
