# Supplementary figures and images for: Mesenchymal stem cell therapy in pulmonary fibrosis: a meta-analysis of preclinical studies
Source: Stem Cell Res Ther. 2021 Aug 18;12:461. doi: 10.1186/s13287-021-02496-2 (PMC8371890; doi:10.1186/s13287-021-02496-2)

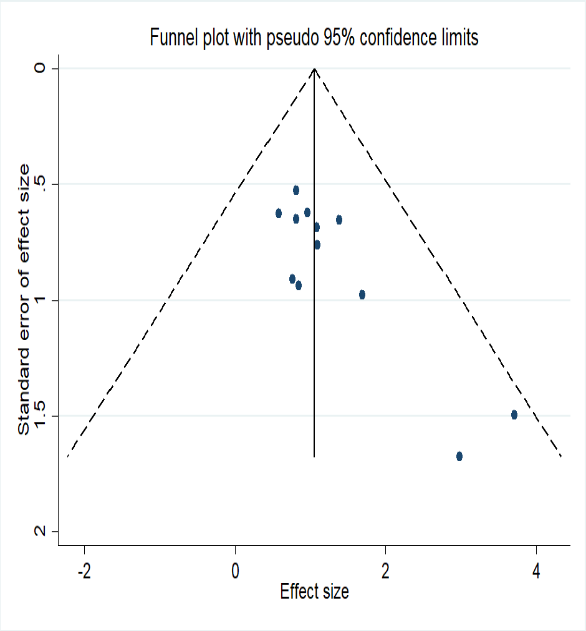

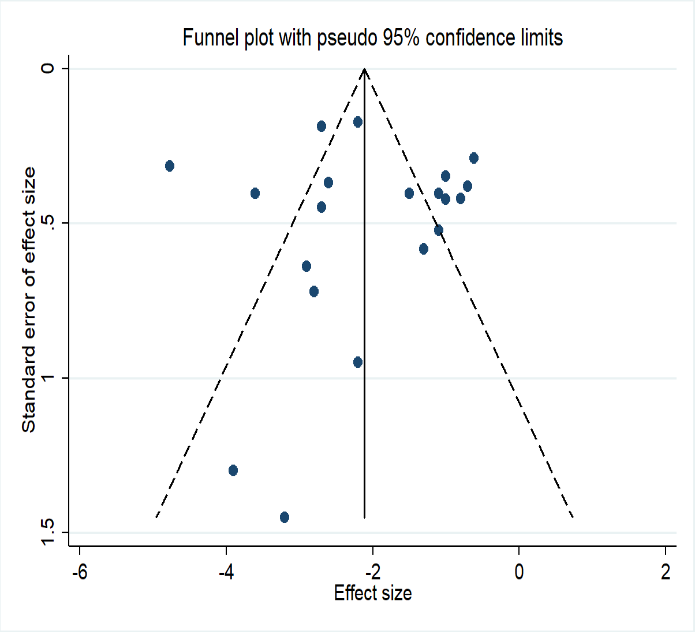


a

b

Fig. S11 Funnel plots for (a) survival rate, (b) pulmonary fibrosis scores.

Supplement: Supplementary file 6 — Additional file 6: Fig. S11. Funnel plots for (a) survival rate, (b) pulmonary fibrosis scores. [file 13287_2021_2496_MOESM6_ESM.docx]
